# Therapeutic Alliances for Optimizing the Management of Patients with Prostate Cancer: SOGUG Multidisciplinary Expert Panel Recommendations

**DOI:** 10.3390/cancers17193208

**Published:** 2025-10-01

**Authors:** Aránzazu González-del-Alba, Claudio Martínez Ballesteros, José Ángel Arranz, Enrique Gallardo, Regina Gironés Sarrió, Fernando López Campos, Jesús Muñoz-Rodríguez, María José Méndez-Vidal, Alfonso Gómez de Iturriaga

**Affiliations:** 1 Department of Medical Oncology, Hospital Universitario Puerta de Hierro-Majadahonda, 28222 Madrid, Spain; 2 Hospital Universitario Puerta de Hierro-Majadahonda, 28222 Madrid, Spain; 3 Hospital General Universitaro Gregorio Marañón, 28007 Madrid, Spain; 4 Department of Oncology, Parc Taulí Hospital Universitari, Institut d’Investigació i Innovació Parc Taulí (I3PT-CERCA), Universitat Autònoma de Barcelona, 08208 Sabadell, Spain; 5 Hospital Universitari i Politècnic La Fe, 46026 Valencia, Spain; 6 Radiation Oncology Department, Hospital Universitario Ramón y Cajal, 28034 Madrid, Spain; 7 Genesis Care Hospital Vithas La Milagrosa, 28010 Madrid, Spain; 8 Department of Urology, Corporació Sanitària Parc Taulí, Institut d’Investigació i Innovació Parc Taulí (I3PT-CERCA), Universitat Autònoma de Barcelona, 08208 Sabadell, Spain; 9 Maimonides Institute for Biomedical Research of Córdoba (IMIBIC), Hospital Universitario Reina Sofía (HURS), Medical Oncology Department, 14004 Córdoba, Spain; 10 Hospital Universitario de Cruces, Biobizkaia Health Research Institute, Basque Country University (UPV/EHU), 48903 San Vicente de Barakaldo, Spain

**Keywords:** prostate cancer, multidisciplinary team, therapeutic alliances, localized disease, locally advanced disease, biochemical recurrence, metastatic disease, geriatric assessment

## Abstract

A group of Spanish experts of different specialties participated in the ENFOCA2 project, promoted by the Spanish Oncology Genitourinary Group (SOGUG), which was designed to provide updated information on current and novel aspects contributing to the optimal care of prostate cancer (PCa) patients. In localized disease, it is important to implement strategic alliances with other institutions for improving adherence to active surveillance in low-risk groups and to explore genetic testing for a better indication of focal therapy. Local control of the disease should be maximized to prevent local failure and biochemical recurrence. In patients with locally advanced disease, with PSMA PET/CT-positive lesions in M0 staging on conventional imaging techniques, therapeutic decisions should be carefully evaluated due to insufficient evidence regarding the gold standard in this setting. In patients with metastatic castration-resistant PCa (mCRPC), assessment of *BRCA* somatic and germline mutations provides prognostic information and familial cancer risk and informs treatment decisions. Combinations of androgen receptor signaling inhibitor (ARSi) agents and poly-ADP ribose polymerase inhibitors (PARPi) are emerging alternatives for advanced PCa. The oldest segment of PCa patients (>70 years of age) may require geriatric assessment to evaluate physical and functional reserves, tailoring treatment to their individual characteristics and circumstances. The concept of a comprehensive multidisciplinary approach together with inter-center and/or inter-specialty therapeutic alliances should be implemented in the routine care of patients with PCa.

## Simple Summary

Prostate cancer (PCa) is a clinically relevant disease because it is one of the most common cancer types in men and one of the leading causes of cancer-related death. Prostate cancer is a heterogeneous disease, although most cases of PCa are slow and often indolent, with a low propensity to progress. In other cases, it may have a clinical pattern characterized by aggressive disease, a high likelihood of recurrence, and progression to an advanced metastatic stage. Although there are available evidence-based guidelines for the management of the different stages of PCa, appropriate knowledge and full application of the novel and advisable strategies in daily practice are still limited. This review is focused on the importance of therapeutic alliances for optimizing the management of PCa patients with localized and locally advanced disease, biochemical recurrence, and advanced stage, especially in case of metastatic castration-resistant PCa (mCRPC) and in patients older than 70 years of age, an increasing segment of the population with special needs due to functional and physical disabilities related to aging. Therapeutic alliances in the framework of a multidisciplinary team approach are the cornerstone of the quality of care and improvement of clinical outcome in the PCa setting.

## 1. Introduction

Prostate cancer (PCa) remains the second most common cancer among men worldwide [1] and is the third leading cause of cancer-related death in men in Europe [2]. PCa is typically a slow-growing disease, with most patients being diagnosed at 65 years of age or older. In its early stages, PCa is usually asymptomatic, and at the time of diagnosis, a large percentage of patients (>80%) have organ-confined disease with a low risk of progression [3]. Locally advanced PCa, involving invasion into some periprostatic tissue and local lymph nodes, is present in 10–15% of patients. Overall, PCa-specific mortality is low, regardless of the treatment approach [4].

However, despite impressive advances in cancer research, the management of patients in daily practice continues to be challenging. The use of diagnostic algorithms may still lead to overdiagnosis, and breaking the compulsory link between diagnosis and active treatment is the recommendable approach to reduce the risk of overtreatment [5]. Decisions related to diagnosis and treatment should be taken based on criteria of a multidisciplinary team (MDT), balancing outcomes and adverse effects of the different treatment pathways and considering the opinions and preferences of the individual patient [6]. Although scientific societies have made extensive efforts to provide updated clinical guidelines for establishing adequate diagnostic and therapeutic approaches for the different stages of PCa [3,7,8,9], the application of recommendations in individual patients may be difficult, not only due to lack of technical resources in hospitals (e.g., diagnostic radioimaging or effective radiation therapy equipment), but also due to local characteristics of healthcare systems, availability of novel systemic agents, and the idiosyncrasy of patients.

To address these complexities, a project (named ENFOCA2) was designed for the final purpose of establishing therapeutic alliances for optimizing the management of patients with PCa. The project integrated the perspectives of various Spanish specialists involved in the care of PCa patients, who discussed different clinically relevant aspects of PCa and proposed their expert consensus opinions to address current challenges. The main topics to be reviewed were selected by participants and included the approach of localized and locally advanced PCa, biochemical recurrence, advanced disease with progression of metastatic hormone-sensitive PCa (mHSPC) to metastatic castration-resistant PCa (mCRPC), inter-center and/or inter-specialty therapeutic alliance in localized and disseminated disease, and geriatric assessment for therapeutic decision-making. For each topic, an overview of the current situation is presented, followed by identification of challenges and proposals to overcome these challenges.

## 2. Therapeutic Alliances for Optimizing Treatment of Localized Disease

### 2.1. Overview of the Current Situation

The approach for patients with localized PCa should consider the prognosis, which is based on individual characteristics and the stratification of risk. Assessment of risk stratification is a crucial aspect to define (a) the need and intensity of treatment, and (b) to support optimal management decisions after careful evaluation of harms and benefits for the individual case, preventing undertreatment and overtreatment [9]. The NCCN^®^ guidelines use risk groups as a framework for stratifying patients with localized disease into ‘very-low-risk group’, ‘low-risk group’, ‘favorable intermediate-risk group’, and ‘unfavorable intermediate-risk group’. These risk groups are extensively used in clinical practice and have been validated, but it is important to consider that there is intrinsic prognostic heterogeneity within each of these groups [10,11,12]. It has been shown that age and high levels of serum prostate-specific antigen (PSA) combined with other risk factors prompt risk reclassification with pathologic upgrading at prostatectomy [13,14].

Intraductal carcinoma (IDC) and large cribriform growth patterns represent aggressive histopathologic features in prostate cancer with significant prognostic implications. Both patterns are significantly associated with extraprostatic extension, seminal vesicle invasion, lymph node metastasis, biochemical recurrence, distant metastasis, and disease-specific death, with pooled odds ratios ranging from 2.55 to 9.84 [15]. IDC is a distinct entity characterized by marked architectural and cytological abnormalities that exceeds high-grade prostatic intraepithelial neoplasia, strongly associated with high Gleason scores and poor clinical outcomes [16]. Studies demonstrated that the presence of any amount of large cribriform pattern or IDC significantly increases the biochemical recurrence risk, with hazard ratios of 2.98 [17] and 4.36 for large cribriform growth specifically [18]. Large cribriform fields, defined as having a diameter at least twice that of adjacent normal glands, show independent prognostic value and are associated with extraprostatic extension and lymph node metastases [18]. The co-occurrence and architectural overlap between cribriform and IDC patterns complicate diagnosis but underscore their shared malignant potential. Consequently, international guidelines now discourage active surveillance in patients with these features, reinforcing their role as exclusion criteria in conservative management strategies.

The future of biomarkers in prostate cancer management is rapidly evolving through genomic, molecular, and AI-driven approaches. High-throughput proteomics and genomics platforms are accelerating biomarker development, with emerging molecular markers, including mRNAs, microRNAs, lncRNAs, and repetitive sequences, expected to enable earlier, more accurate diagnosis in precision medicine contexts [19,20]. Current molecular biomarkers encompass serum markers like PHI and 4K score, urine-based tests including PCA3 and SelectMDx, and tissue-based assays such as Oncotype DX and Decipher [21]. These synergistic advances in AI and sequencing technologies are facilitating clinical decision-making and improving personalized prostate cancer management.

#### 2.1.1. Low-Risk PCa

Active surveillance has been the preferred option for patients in the very-low-risk group and for most patients in the low-risk group and is currently considered the standard of care. Observation is the recommended approach in patients with life expectancy of <10 years. However, selecting the correct patient profile for active surveillance is essential to prevent undertreatment. Moreover, active surveillance strategies for patients with low-risk PCa are not clearly defined, and for men electing for active surveillance there is no consensus regarding the composition and optimal frequency of follow-up testing (e.g., magnetic resonance imaging (MRI), prostate biopsy, PSA testing (velocity and density), or minimum threshold tumor volume). A recent white paper by the Genitourinary Pathology Society and the International Society of Urological Pathology, which emphasizes the limitations of biopsy-based diagnosis and the need for a multidisciplinary approach to define indolent prostate cancer, supports the integration of clinical, radiological, and pathological criteria to optimize patient selection for active surveillance [22].

In a study that evaluated the cost-effectiveness of surveillance strategies based on a mathematical model for low-risk PCa diagnosis, biopsy-guided decisions according to MRI findings performed annually were associated with a reduced the number of biopsies, preserving quality of life and life expectancy [15]. Also, biopsy in lesions with Prostate Imaging Reporting and Data System (PI-RADS) scores of ≥ 4 was likely the most cost-effective strategy for patients younger than 70 years [23].

Radical therapy with radical prostatectomy as well as radiation therapy are still options for low-risk PCa, although there is a risk of overtreatment, and the 15-year follow-up publication from the ProtecT trial [4] provided evidence of the reduction of local progression, incidence of metastases, and long-term use of androgen deprivation therapy (ADT) associated with radical treatment. However, radical prostatic surgery did not affect mortality and was associated with important adverse effects [4]. Focal therapy includes several energy sources, such as photodynamic therapy, high-intensity focused ultrasound (HIFU), laser, cryotherapy, brachytherapy, or irreversible electroporation (IRE), but robust evidence in favor of focal therapy is still lacking [24]. Other alternatives, such as nutraceutical products, physical activity, or immunotherapy, have been explored in low-risk localized PCa but their efficacy remains limited [25,26,27].

#### 2.1.2. Favorable Intermediate-Risk PCa

Patients in this subgroup could be distinct candidates for active surveillance in the presence of PSA levels < 10 ng/mL, Gleason score ≤ 3 + 4 (<10%), stage ≤ cT2a, and ≤3 positive cores (<50% positive biopsy). Prostate surgery and radiation therapy are generally the treatment of choice. Nomograms can be used for the prediction of lymph node invasion, and new minimally invasive surgical procedures help to preserve functional outcomes. In the context of radiation therapy, several options are available, including brachytherapy, stereotactic body radiation therapy (SBRT), and hypofractionated radiotherapy. However, further evaluation to determine whether any one of these techniques is superior to the others is necessary [28]. Focal therapy represents a feasible and acceptable approach for selected patients [29], although its use should still be limited to clinical trials.

#### 2.1.3. Unfavorable Intermediate-Risk PCa

Patients in the unfavorable intermediate-risk group are not candidates for watchful waiting, and 10–15% of patients with favorable intermediate-risk (with ISUP 2 disease re-biopsy and >3 positive cores (>50% positive biopsy)) undergoing active surveillance progress to the unfavorable group [30]. The standard approach regarding extended lymph node dissection and the preservation of neurovascular bundles in radical prostatectomy remains unclear, as does the systematic indication and optimal duration of ADT. The combination of external beam radiotherapy and high-dose-rate brachytherapy is considered an optimal radiotherapy treatment modality for suitable candidates [31]. Focal therapy, accompanied by mapping biopsies, is a therapeutic alternative for individual patients with unfavorable intermediate-risk prostate cancer [32]. However, careful patient selection is crucial, as inappropriate selection may not only reduce treatment effectiveness but also increase toxicity, potentially worsening side effects rather than minimizing them.

### 2.2. Challenges

- Standardize the definitions of very-low- and low-risk localized PCa groups.
- Standardize the strategy for the frequency of MRI and biopsy.
- Monitor the application of active surveillance protocols in real-world practice, ensuring patients’ adherence.
- Gather data from a watchful waiting approach in large series of elderly patients.
- Select the best candidates for focal therapy and the feasibility of radical surgery as rescue therapy for focal ablation.

### 2.3. Recommendations

- Implementation of strategic alliances with other institutions for improving adherence to active surveillance protocols in low-risk groups.
- International guidelines discourage active surveillance in patients with IDC and large cribriform growth patterns, reinforcing their role as exclusion criteria in conservative management strategies.
- Explore genetic testing for improving the selection of patients for focal therapy.
- In the unfavorable intermediate-risk category, it is important to define what is a “sufficient” lymph node dissection and to incorporate new imaging techniques for focal radiotherapy boost.
- Evaluation of the sum of ADT and radiotherapy in the real-world setting.
- Selection of candidates to ablation therapies should be based on findings of advanced imaging studies and genetic testing.

## 3. Therapeutic Alliances for Optimizing Treatment of Locally Advanced Disease

### 3.1. Overview of the Current Situation

In patients with high-risk or very-high risk PCa, the initial and adjuvant therapy depend on the expected patient survival with external beam radiation therapy (EBRT) plus ADT or radical prostatectomy and pelvic lymph node dissection as the initial approach for patients with >5 years life expectancy or symptomatic patients, and observation or ADT or EBRT for asymptomatic patients or those with ≤5 years life expectancy [9,33]. For any tumor (T) stage and lymph node (N)-positive disease (N1), the initial treatment includes EBRT plus ADT plus abiraterone (preferred option) or radical prostatectomy and pelvic lymph node dissection in patients with >5 years life expectancy or symptomatic patients, whereas observation or ADT or best supportive care is indicated in asymptomatic patients and those with ≤5 years life expectancy [9]. However, there is limited evidence that radical prostatectomy plus pelvic lymph node dissection is beneficial in the case of node-positive disease. This approach should be restricted to patients with >10-year life expectancy and resectable disease and would be recommendable in the framework of a clinical trial or planned multimodality approach. Guidelines for radical and palliative treatment in patients with locally advanced disease are shown in Table 1 [33].

Multimodal treatments, including radical prostatectomy, radiation therapy, and hormone therapy, are primary therapeutic modalities in the management of PCa patients with high-risk and very-high-risk disease, but in areas where the evidence is inconclusive or controversial, multidisciplinary collaboration is crucial for supporting shared decision-making [34]. Also, the benefits of systemic therapy combinations (e.g., docetaxel or an androgen receptor signaling inhibitor) for high-risk and/or unfavorable PCa as part of primary definitive therapy are unclear [35].

In an investigation of the impact of ADT sequencing for men receiving ADT with prostate-only radiotherapy (PORT) or whole-pelvis radiotherapy (WPRT) from 12 randomized trials with a total of 7409 patients, concurrent/adjuvant ADT was associated with a significant interaction with field size and improved metastasis-free survival, distant metastasis, prostate cancer-specific mortality, and overall survival, compared with neoadjuvant/concurrent ADT in the PORT group [36]. According to these findings, when short-term ADT is indicated in combination with PORT, it is concluded that the standard of care should be concurrent/adjuvant ADT [36].

In a pooled analysis of two subpopulations of two randomized controlled phase 3 trials of the STAMPEDE platform protocol, patients with high-risk nonmetastatic PCa who received abiraterone and prednisone for two years added to ADT had better metastases-free survival and overall survival compared to ADT alone. Awaiting the results of randomized clinical trials, this combination, as well as local radiotherapy if indicated, might be considered for nonmetastatic prostate cancer with high-risk features [37].

On the other hand, in relation to controversy about the extension of lymph node dissection during prostatectomy, the pattern of lymphatic drainage of the prostatic gland is complex, and the identification of the sentinel node is not able to replace extended pelvic lymph node dissection [38].

The detection and management of PCa have been remarkably improved by recent advances in imaging techniques. Prostate-specific membrane antigen (PSMA)-based positron-emission tomography/computed tomography (PSMA PET/CT) is one of these innovations, currently representing an accurate technique for diagnosing and monitoring PCa. In a recent systematic review and meta-analysis, the sensitivity and specificity of PSMA PET/CT for localized PCa were 71.0% and 92.0%, respectively [39]. However, the role of PSMA PET/CT in different PCa scenarios is still undefined.

### 3.2. Challenges

- Evaluation of the contribution of PSMA PET/CT in staging of locally advanced PCa, particularly in patients with M0 on conventional imaging studies and PSMA PET/CT-positive lesions.
- To assess the real risk of metastatic disease to prevent the risk of undertreatment or overtreatment.
- Adequate surgical planning is crucial to avoid residual disease in patients with locally advanced PCa.
- To define postsurgical criteria for the use of neoadjuvant radiotherapy vs. early rescue.
- To establish the best scheme of radiotherapy plus ADT in the individual patient and neoadjuvant strategies (ADT, second-generation antiandrogens).
- To assess the advantages of PSMA-guided surgery, robotic surgery, and preoperative and postoperative PSMA.

### 3.3. Recommendations

- External beam radiation therapy (EBRT) or EBRT plus brachytherapy boost and ADT for 2–3 years is the recommended radiotherapy scheme.
- PSMA PET/CT is recommended in high- and very-high-risk PCa and should not be used in the intermediate-risk group.
- In the presence of PSMA PET/CT-positive lesions in M0 staging on conventional imaging techniques, therapeutic decisions should be carefully evaluated due to insufficient evidence regarding the gold standard in this setting.
- Surgical treatment should be indicated in patients with >10 years of life expectancy and in the framework of multimodal therapy.
- Abiraterone–prednisone added to 2-year ADT improves outcomes in cN1 patients treated with radiotherapy as well as in cN0 patients with PSA > 40 ng/mL, Gleason score 8–10, and T3/4 (SAMPEDE trial criteria).

## 4. Therapeutic Alliances for Optimizing Treatment of Biochemical Recurrence

### 4.1. Overview of the Current Situation

Distant metastasis-free survival has been established as a reliable surrogate for overall survival in PCa [40]. Biochemical recurrence (BCR), identified by elevated PSA levels, is a commonly used parameter in the evaluation of outcomes after curative treatments (radical prostatectomy or radiotherapy). The primary mechanism for the development of distant metastasis occurs after an initial relapse-free period, likely originating from the presence of metastatic disease that was undetected when the diagnosis of PCa was established. This phase, which typically occurs within the first four years after treatment completion, emphasizes the importance of accurate initial staging and systemic therapy. Subsequently, a second wave of distant metastases emerges, primarily linked to local failure. A pooled analysis of data from 18 randomized trials with 12,533 patients with PCa (6245 intermediate risk and 6288 high risk) showed that 81% of distant metastases occurred in clinically relapse-free scenarios [41]. Local failure was found to be significantly associated with overall survival, PCa-specific survival, and distant metastasis-free survival, particularly among high-risk patients. In the absence of local failure, the risk of transitioning to a PCa-specific death was notably lower, as compared to the presence of local failure [41].

**Figure 1:**
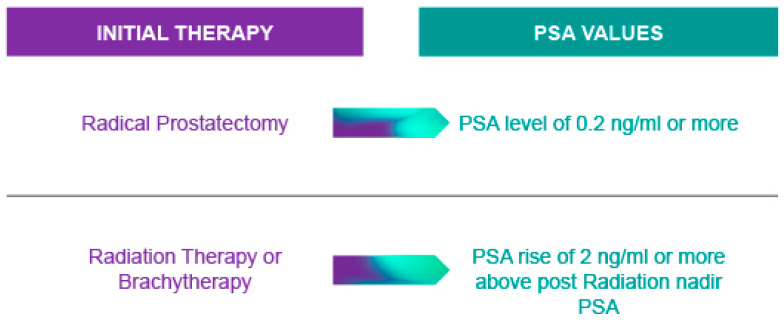
Definition of biochemical recurrence (BCR) after initial curative treatment.

There is a high prevalence of BCR after local treatment. BCR develops in about 35% of patients treated with radical prostatectomy, and is an independent factor associated with the risk of survival, including overall survival, PCa-specific survival, and survival-free metastatic disease [42]. Men with a high tumor grade and PSA rising shortly after surgery or radiotherapy are at the highest risk of mortality [42]. A standardized definition of BCR (Figure 1) is essential given that the risk of subsequent metastatic disease progression may vary greatly depending on the PSA criterion used.

The European Association of Urology (EAU) stratified the BCR risk into two groups (low and high risk) according to the aggressiveness of the tumor and doubling time of PSA [43]. EAU low-risk BCR is defined as PSA doubling time (PSADT) > 12 months and ISUP grade < 4, and EAU high-risk BCR is defined as PSADT < 12 months or ISUP grade 4–5 [43].

Given that BCR is a very relevant factor increasing the risk of metastatic disease and death in patients with prognostically unfavorable features, the selection of an adequate initial treatment (local, systemic, or both) is of utmost importance. The DADSPORT Meta-analysis Collaboration conducted a systematic review and meta-analysis aiming to evaluate the results of hormone treatment in men treated with radiotherapy following radical prostatectomy for localized PCa and without regional or distant metastasis [44]. Pooled results from different trials (GETUG-AFU 16, NRG/RTOG 9601, NRG/RTOG 0534, and RADICALS-HD trials) were analyzed. The active arm included hormone treatment administered during 6 or 24 months, and no hormone therapy was given in the control arm. After a median follow-up of ≥ 8 years, there was no clear improvement in overall survival between the use or non-use of hormone therapy (hazard ratio (HR) 0.89, 95% confidence interval (CI) 0.77–1.03), as well as over 6 months and 24 months duration of treatment. However, 6-month hormone therapy improved metastases-free survival compared to no hormone therapy (HR 0.82, 95% CI 0.70–0.96, *p* = 0.013) [44]. However, updated results of the effects of hormone therapy on oncological outcomes, including final network meta-analysis incorporating all direct and indirect evidence, as well as effects by pre-defined patient subgroups, are waiting to be reported.

In a retrospective review of patients with PSMA-PET imaging for BCR following radical prostatectomy with PSA ≤ 2.0 ng/mL, 53% of patients had PSMA-avid disease (38% lesions outside the pelvis, 50% lesions confined to the pelvic lymph nodes and prostate bed, and 18% lesions in the prostate bed only) [45]. Therefore, PSMA-avid disease would have been missed by standard salvage radiation fields in nearly one-third of patients. Moreover, in data from the proMAS trial, a multicenter, two-arm, randomized study of 302 men with biopsy-proven PCa and high-risk features, PSMA PET-CT had a 27% greater accuracy than CT and bone scanning for detecting pelvic node and distant metastases [46]. Also, PSMA PET showed a high predictive value (64.5%) for a three-year freedom from progression in men with BCR after radical prostatectomy undergoing salvage radiotherapy [47]. PSMA PET-CT has emerged as a powerful diagnostic tool that can identify biochemically recurrent PCa at low PSA levels that is still potentially curable, which has a high management impact.

Data of the EMBARK phase 3 randomized study of enzalutamide or placebo plus leuprolide acetate and leuprolide monotherapy in high-risk PCa patients with BCR showed that the combination of enzalutamide/leuprolide vs. leuprolide alone demonstrated a significant improvement in metastasis-free survival (HR 0.42, 95% CI 0.31–0.61, *p* < 0.0001), overall survival in the interim analysis (HR 0.59, 95% CI 0.38–0.90, *p* = 0.0142), and time to PSA progression (HR 0.07, 95% CI 0.03–0.14, *p* < 0.0001) [40,41]. Since these results, enzalutamide in combination with ADT has been indicated in patients with high-risk BCR with a PSADT < 9 months with no evidence of metastasis on conventional imaging, and who are not candidates for local salvage therapy [48,49].

Genomic PCa tests, such as the Decipher genomic risk score, are also promising tools that may aid in managing PCa patients throughout the continuum of care and delivering appropriate treatment at an individualized level [50,51]. Combined cell-cycle risk scores and cell-cycle progression are other biomarkers that improved risk discrimination for predicting metastases and disease-specific survival after radical prostatectomy, helping to identify patients at greatest risk of treatment failure who might benefit from earlier intervention [52].

An ongoing phase 3 study (NCT05050084) uses the Decipher risk score to assess whether men with unfavorable intermediate-risk PCa and lower Decipher scores (<0.40) treated with radiotherapy alone instead of six-month ADT + radiotherapy experienced non-inferior rates of distant metastasis (de-intensification study), or whether men with higher Decipher scores (≥40) will have a superior metastasis-free survival through treatment intensification with darolutamide added to the standard of radiotherapy plus six months of ADT (intensification study). The EA8191/INDICATE phase 3 trial (NCT 04423211) evaluated whether enhanced therapy (apalutamide in combination with abiraterone + prednisone) added to the standard of care (SOC; prostate radiation therapy and short-term ADT) is more effective compared to SOC alone in PCa patients with BCR after radical prostatectomy, as well as the benefit of adding metastasis-directed radiation to enhanced therapy (apalutamide in combination with abiraterone + prednisone) in PCa patients with BCR and metastasis outside the pelvis shown by PET imaging.

### 4.2. Challenges

- Improvement of local control of the disease as much as possible to prevent local failure.
- Definition of clear criteria for defining BCR based on the PSA level combined with the ISUP grade and doubling time of the PSA level.
- Selection of an adequate initial treatment (local, systemic, or both).
- Implementation of PSMA-PET imaging in patients with BCR, especially at low PSA levels for detecting pelvic nodal and distant metastases.
- Application of genomic-based risk tools to identify patients at greatest risk of treatment failure who might benefit from earlier intervention.

### 4.3. Recommendations

- Importance of maximizing the chances of a cure by intensification of treatment before failure.
- Investigation of the effects of changes of treatment based on next-generation imaging findings.
- Genomic classifiers could make it easier to choose when and how treatment intensification is required.
- The implementation of a multidisciplinary team approach and treatment individualization in clinical practice is essential to improve oncological outcomes of men with BCR of PCa.

## 5. Therapeutic Alliances for Optimizing Treatment in the Sequence of Advanced Disease

### 5.1. Overview of the Current Situation

**Figure 2:**
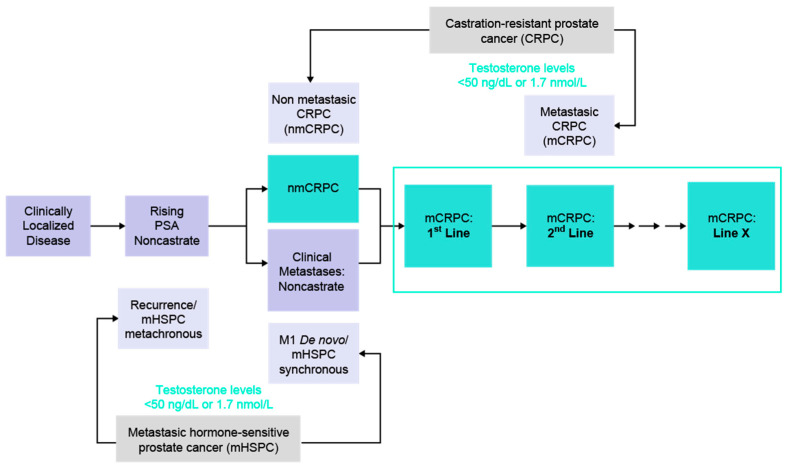
Pathways of the continuum of PCa from localized disease to advanced stages and the corresponding lines of treatment.

**Figure 3:**
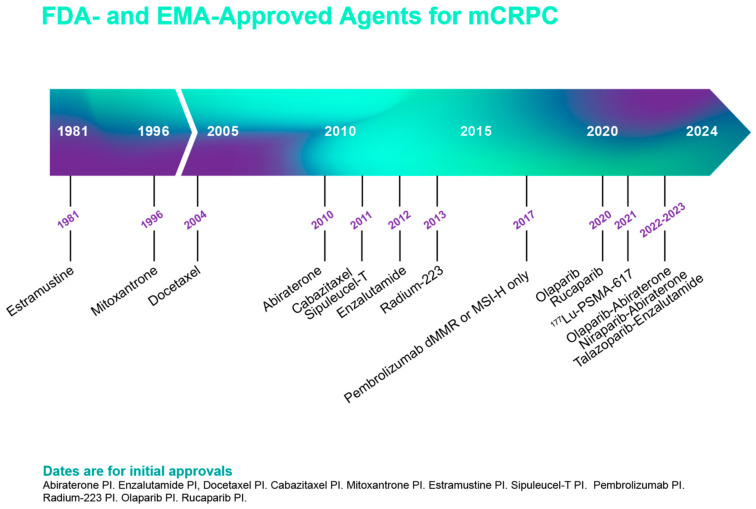
Approved agents by the Food and Drug Administration (FDA) for the treatment of men with metastatic castration-resistant PCa (mCRPC) (Abi: abiraterone; BRCA mut: BCRA-mutated).

Recently, there has been an annual increase of about 5% in the incidence of advanced PCa including metastatic hormone-sensitive prostate cancer (mHSPC) [53]. Progression to metastatic castration-resistant PCa (mCRPC) is the usual cause of death, but sequencing of the disease is evolving due to several factors, including the effect of the approval of new classes of drugs for use in various disease states (Figure 2 and Figure 3).

The strategies of treatment in advanced PCa of first line and second and subsequent lines and the results obtained in randomized studies [54,55,56,57,58,59,60] are shown in Table 2. Patients enrolled in clinical trials, however, are not representative of patients with mCRPC attended in daily practice.

In an attempt to define the most effective strategy for improving overall and progression-free survival, several studies have evaluated combinations and sequences of treatments in patients with mCRPC. It has been shown that the use of enzalutamide and abiraterone in sequence is of limited benefit due to cross-resistance between these agents, and in case of PSA progression during enzalutamide monotherapy, treatment with enzalutamide in combination with abiraterone should not be continued [61]. In another clinical trial, treatment with cabazitaxel in patients with mCRPC previously treated with docetaxel who had disease progression within 12 months while receiving abiraterone or enzalutamide was associated with longer imaging-based progression-free survival and overall survival, as compared with treatment with enzalutamide (in those who had previously received abiraterone) or abiraterone (in those who had previously received enzalutamide) [62]. In patients with mCRPC and qualifying alterations in homologous recombination repair genes who showed progression of disease during previous treatment with next-generation hormonal agents, the use of olaparib was associated with a significantly longer overall survival, as compared with enzalutamide or abiraterone/prednisone [63]. On the other hand, in the presence of alterations of the *BRCA1*, *BRCA2*, or *ATM* genes and progression of disease following treatment with a second-generation androgen receptor pathway inhibitor (ARPI), the administration of rucaparib, an inhibitor of poly(ADP-ribose) polymerase (PARP), prolonged the duration of imaging-based progression-free survival (11.2 months), as compared to the control arm (6.4 months; no medication and includes docetaxel chemotherapy or other ARPi) [64]. In addition, the analysis of *BRCA* and *ATM* subgroups showed significantly longer duration of imaging-based progression-free survival in the rucaparib group than in the control group in both *BRCA* and *ATM* subgroups, but the overall survival was more favorable for rucaparib vs. control in the *BRCA* subgroup only [64].

Lutetium-177 (^177^Lu)-PSMA-617 is a radioligand therapy that delivers beta-particle radiation to PSMA-expressing cells, and it is well-known that PSMA is highly expressed in mCRPC. In an open-label phase 3 trial, the radioligand ^177^Lu-PSMA-617 was evaluated in mCRPC patients previously treated with at least one androgen receptor pathway inhibitor and one or two taxane regimens and who had PSMA-positive gallium-68 (68Ga)-labeled PSMA-11 PET-CT [56]. After a median follow-up of 20.6 months, ^177^Lu-PSMA-617, as compared to SOC, prolonged imaging-based progression-free survival (8.7 vs. 3.4 months, *p* < 0.001) and overall survival (15.3 vs. 11.3, *p* < 0.001) [65]. On the other hand, in a randomized phase 2 study, ^177^Lu-PSMA-617, as compared with cabazitaxel in patients with mCRPC (previous treatment with docetaxel with ARTA was allowed), led to higher PSA responses, greater benefit in quality of life (QoL), a favorable safety profile, and better PFS [66]. On the basis of these findings, Lu-PSMA-617 was proposed as a potential alternative to cabazitaxel with a better safety profile in patients with mCRPC treated with prior docetaxel [66]. Rad223 is an option for patients with mCRPC with bone metastases without visceral involvement (ALSYMPCA study) [60]. In the ERA trial [67] the combination with abiraterone was detrimental in terms of OS, with an increase in fractures in the combination arm. More recently, it has been shown that the combination of Rad223 with enzalutamide provided benefit in PFS and OS compared to enzalutamide alone in first-line mCRPC patients, in which the use of bone protective agents was mandatory [68].

Androgen receptor signaling inhibitor (ARSi) agents (and poly-ADP ribose polymerase inhibitors (PARPi)) are emerging therapeutic options for advanced PCa. Evidence of the important role played by a somatic/germline homologous recombination repair (HRR) gene mutation (mainly in *BRCA2*) in the progression of the disease is one factor supporting the rationale of PARPi in PCa. At the molecular level, maximal androgen receptor blockade via an ARSi in combination with a PARPi has a synergistic effect, leading to synthetic lethality in both HRR-mutated and HRR-non-mutated PCa patients [69]. Also, selective targeting of PARP-2 may provide an alternative therapeutic approach for androgen receptor inhibition by disruption of FOXA1 function, which may be beneficial to patients, independent of their DNA-repair deficiency status [70]. In the mCRPC setting, recent phase 3 trials have evaluated the use of PARPi in combination with an ARSI. In all trials, PROpel (olaparib and abiraterone acetate/prednisone) [71], TALAPRO-2 (talazoparib and enzalutamide) [72], and MAGNITUDE (niraparib and abiraterone acetate/prednisone) [73] included both HRR-mutated and HRR-non-mutated patients and showed a marked increase in radiographic progression-free survival, time to first subsequent therapy or death, and a trend toward better overall survival with the use of PARPi in combination with an ARSi, compared to an ARSi alone in the HRR-mutated population, with the greatest benefit in the *BRCA*-mutated patients. In the TALAPRO-2 and PROpel trials, improvements in radiographic progression-free survival were also shown in non-HRR-mutated patients.

In nonmetastatic castration-resistant prostate cancer (nmCRPC), results of the ARAMIS phase 3 trial showed that darolutamide, as compared with placebo, was associated with a significantly prolonged metastasis-free survival and a risk of death significantly lower, by 31%, in the darolutamide group than in the placebo group [74]. These favorable results were also reported in the analysis of the subgroup population of Spanish patients [75]. In the SPARTAN phase 3 trial, apalutamide vs. placebo showed a 22% decrease in the hazard of death [76]. Finally, in nmCRPC patients with a rapidly rising PSA level, results of the PROSPER phase 3 trial showed a 27% lower risk of mortality and longer median overall survival as compared with placebo for treatment with enzalutamide plus ADT [77].

The treatment of patients with mHSPC has also been changed from the introduction of docetaxel in 2015 to the more recent triple-combination therapies. In the randomized phase 3 ARASENS trial [78], patients were assigned to treatment with darolutamide or matching placebo, both in combination with androgen-deprivation therapy and docetaxel. The risk of death was significantly lower by 32.5% in the darolutamide combination group. In men with de novo mHSPC, the PEACE-1 trial [79] demonstrated that combining ADT and docetaxel with abiraterone acetate/prednisone improved both overall survival and radiographic progression-free survival. However, in patients with low tumor volume and low metastatic burden, adding radiotherapy to standard treatment plus abiraterone improves radiographic progression-free survival and castration-resistant survival, but not overall survival [79].

In biomarker-selected populations of patients with metastatic castration-sensitive prostate cancer (mCSPC), there are a number of ongoing randomized phase 3 clinical trials to assess different treatment strategies, including niraparib/placebo added to abiraterone acetate/prednisone with ADT in the AMPLITUDE trial (NCT04497844), talazoparib plus enzalutamide vs. placebo plus enzalutamide with ADT in the TALAPRO-3 trial (NCT04821622), saruparib added to physician’s choice new hormonal agents (NHAs; abiraterone acetate, darolutamide, or enzalutamide) relative to placebo plus physician’s choice NHAs in the EvoPAR-Prostate01 trial (NCT06120491), ^177^Lu-PSMA-617 plus SOC vs. SOC alone in the PSMAddition trial (NCT04720157), and capivasertib plus abiraterone vs. placebo plus abiraterone with ADT in the CAPItello-281 trial (NCT04493853).

### 5.2. Challenges

- Assessment of *BRCA* somatic and germline mutations in patients with mCRPC to have available prognostic and predictive information as well as familial cancer risk.
- To determine the optimal agent combination or sequence in the individual patient based on a multidisciplinary team consensus.
- Definition of the most adequate strategy for non-HRR mCRPC patients in the first-line setting (ARSi monotherapy or combination).
- Integration of new-generation imaging techniques into clinical practice for diagnosis, patient selection, treatment, and disease monitoring.

### 5.3. Recommendations

- Patients with advanced PCa require an integral approach established by a multidisciplinary team from the beginning.
- It is essential to select the most effective first-line treatment because subsequent strategies depend on this choice.
- The continuous emergence of therapeutic advances that impact survival adds greater complexity to the design of an overall strategy for the care of PCa patients with advanced disease.
- Access to therapeutic innovation must be considered in all scenarios.

## 6. Inter-Center and/or Inter-Specialty Therapeutic Alliances for Optimizing Treatment in Localized and Disseminated Disease

### 6.1. Overview of the Current Situation

Despite impressive advances in research and development of clinical practice guidelines for all stages of PCa, the optimum management for an individual patient in daily practice is not well defined. Differences in professional knowledge, healthcare systems, organizational policies, media, cultural beliefs, and values are challenging to ascertain but are important drivers in the real-world variability for optimizing the care of patients with PCa.

Various studies have provided evidence of inter-specialty divergences. In a study that evaluated the variability in accuracy of PCa segmentation among radiologists, urologists, and scientists, less experienced participants appeared to under-segment models and underestimate the size of prostate tumors, although radiologists also showed high variability [80]. A nationwide retrospective study in The Netherlands assessing prostate needle biopsy reports of 35,258 patients graded by 40 pathology laboratories showed a considerable variation between and within laboratories (over half of the laboratories graded significantly different from the national mean) and this probably affects treatment strategy and prognosis assessment of PCa patients [81]. In a study of 26 centers with members in the Society of Abdominal Radiology Prostate Cancer Disease-focused Panel, in which 3449 men with 5082 lesions were evaluated, the estimated positive predictive value of the Prostate Imaging Reporting and Data System (PI-RADS) was 35% for a PI-RADS score ≥ 3 and 49% for a PI-RADS score ≥ 4 and varied widely across centers [82]. Moreover, variability in training datasets, algorithms, and evaluation criteria are recognized as current limitations of artificial intelligence (AI) models for detecting prostate cancer on radiology images (MRI and ultrasound imaging) and on histopathology images of biopsy tissue [83].

The diagnosis, staging, and treatment of PCa require a comprehensive approach that includes diagnosis (clinical, laboratory, imaging, biopsies, and histopathology), surgical, radiotherapy, medical, and supportive treatment, studies of hereditary familial cancer, psychological, emotional, and social support, treatment of sequelae and iatrogenesis, and palliative and supportive care. Integrated delivery systems are characterized by organized, collaborative networks that link healthcare providers who are clinically and fiscally accountable for PCa populations across the continuum of care [84]. Integrated delivery systems are considered as a focus in evidence-based healthcare systems that are fully coordinated, aiming to manage and obtain improved clinical outcomes. It has been shown that integrated PCa centers based on multi-specialty consolidation, especially urology–radiation oncology practice, increases the use of intensity-modulated radiation therapy (IMRT) and decreases ADT and prostatectomy [85]. Also, prostate cancer center (PCC) certification programs with fulfilment of requirements contribute to establishing multidisciplinary teams over time and assure the provision of high-quality PCa care [86].

**Figure 4:**
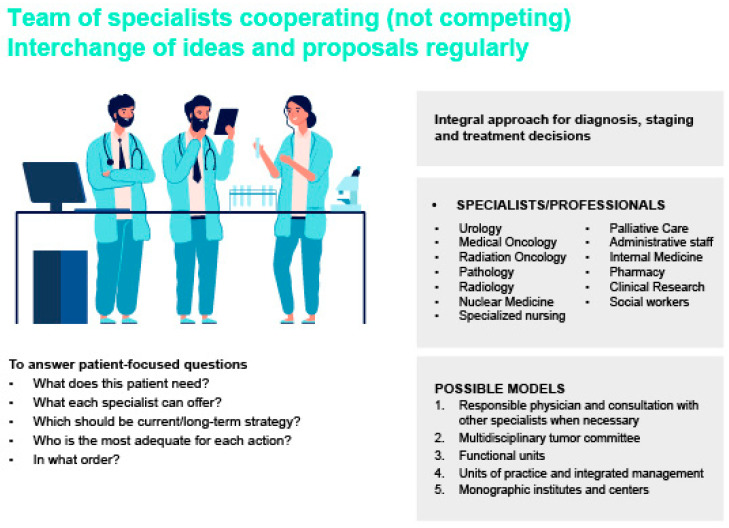
Components of an integral perspective in the management of patients with PCa.

The implementation of PCa multidisciplinary teams (MDTs) in clinical practice plays a key role along the four phases of the patient journey—initial presentation, diagnosis and staging, treatment decision, and follow-up and monitoring—particularly in addressing tests to be completed, interpreting the results of imaging and genetic testing, and choosing adequate treatment options. The importance of an integral approach in the management of PCa patients is illustrated in Figure 4.

Unfortunately, there are still many PCa patients who do not receive a comprehensive multidisciplinary approach tailored to their current needs and overall individualized strategy. However, in response to the need for high-quality management of PCa, the European Prostate Cancer Centers of Excellence (EPCCE) was a novel proposal developed by the European Association of Urology Prostate Cancer Centre Consensus Meeting [87]. A task force of experts identified general criteria to define the EPCCE in the three fields of clinics, research, and education (Table 3). Moreover, the inclusion of a quality control approach represents the novelty that supports the excellence of these centers.

The European Cancer Organisation (ECCO) provides ECCO Essential Requirements for Quality Cancer Care (ERQCC), written by experts representing all disciplines involved in cancer care in Europe, aiming to give oncology teams, patients, policymakers, and managers an overview of essential care throughout the patient journey. In the case of PCa, the recommendations include that care must only be carried out in prostate cancer units or centers that have a core MDT and an extended team of health professionals, paying particular attention to patient-centered pathways from diagnosis, to treatment, to survivorship [88].

### 6.2. Challenges

- To understand and internalize the concept of a comprehensive multidisciplinary approach.
- To identify and solve the barriers to multidisciplinary work, such as the lack of physical infrastructure and administrative support, and the absence of consensus protocols to avoid repeated discussions about differences in therapeutic recommendations, and to find solutions for conflicts of competencies and duplications.
- To improve the collaboration of medical management and hospital administration to promote the creation of MDT infrastructures at various levels of care.
- Creation of supra-structures favoring inter-hospital collaborative networks, development and implementation of novel special techniques, and participation in multicenter clinical trials.

### 6.3. Recommendations

- Establishment of dynamics of respect and interdisciplinary recognition.
- Ensuring a multidisciplinary approach in proposals and discussions, and specialization in the execution of decisions.
- Development and implementation of clear norms regarding organization, hierarchies, and responsibilities.
- Protocols should be collaborative, and each decision should place greater value on the opinion of the specialist most knowledgeable in that specific aspect of the MDT. Periodic updating of protocols is indispensable.
- Expand the scope of supra-structures to include inter-center and cooperative groups.

## 7. Therapeutic Alliances for Optimizing Geriatric Assessment in Therapeutic Decision-Making

### 7.1. Overview of the Current Situation

World population prospects 2024 of the United Nations [80] indicate that by the late 2070s, the number of persons at ages 65 years and higher globally is projected to reach 2.2 billion, surpassing the number of children (under age 18), and by the mid-2030s, it is projected that there will be 265 million persons aged 80 years or older, more than the number of infants (1 year of age or less). Moreover, even in countries with rapidly growing populations and relative youthful segments, the proportion of individuals aged 65 years or older is expected to rise over the next three decades [89]. This situation will directly affect the number of older men with PCa, particularly the percentage of patients aged 75 years or more at the time of diagnosis. In fact, patients with a high burden of comorbid diseases and older age are not proportionally represented in clinical trials. Therefore, despite the incidence of PCa peaking in older age, it is unknown whether older men have the same benefit from the treatment strategies used in younger counterparts.

Given that older patients tend to have more medical comorbidities and less physical functional reserve, they may benefit from tailored approaches to treatment. However, functional reserve and frailty are heterogeneous processes that are not fully coincident with or defined by chronological age [90].

The risk of making decisions based only on chronological age may involve undertreatment and overtreatment, implies ageism, and undermines the patient’s ability to participate in decision-making. The International Society of Geriatric Oncology (SIOG) reported an expert consensus with updated guidelines on the management of PCa in men aged > 70 years [91], the main actions of which are summarized in Table 4.

Geriatric assessment refers to assessing domains where older patients frequently have needs to characterize their overall health status. The domains of the geriatric assessment are functional status, mobility, cognition, nutritional status, mental health, comorbidities, polypharmacy, and social support. Several shorter screening tools are available that can be used to screen patients for those most likely to benefit from a complete geriatric assessment. The Geriatric 8 (G8) screening tool is validated and highly referenced questionnaire in geriatric oncology, which encompasses eight questions that take 4–5 min to complete and covers screening for food intake, weight loss, mobility, neuropsychological conditions, body mass index, polypharmacy, self-assessed health status, and age. The G8 scores range from 0 to 17, with lower scores associated with increasing frailty. In patients with cancer, scoring below 14 has an 85% sensitivity and 64% specificity for detecting frailty [92]. There is strong evidence of the improvement in prognosis and risk stratification due to the use of G8 and other assessment tools (e.g., Mini-Cog for referral to neuropsychiatric testing), as well as providing patient-centered treatment with effective communication, and individualizing care in order to prevent undertreatment and excess toxicity [93].

Older adults with PCa have many integrated domains that may require intervention when treating their oncological disease, with special attention to optimizing bone health and reducing frailty to increase tolerance to both ADT and chemotherapy. Also, it is important to consider other aspects, such as non-cancer life expectancy, goals and values, mobility/transportation barriers, and social support, at the time of development of patient-specific treatment plans.

### 7.2. Challenges

- Improvement of training in oncogeriatrics and inclusion of geriatricians in multidisciplinary teams.
- Increase representation of older populations in clinical trials.
- Change of healthcare systems focused on acute diseases and sometimes inadequate for the care of patients with chronic conditions.
- Adequate assessment of comorbidities that may interfere with the diagnostic and therapeutic approach of PCa.
- Proper evaluation of polypharmacy and risk of drug interactions.
- Consideration of social support and special needs of the oldest old.

### 7.3. Recommendations

- Implementation of geriatric assessment tools (e.g., G8 and 6-min walk test) to reduce the screening time.
- Implementation of functional multidisciplinary units to prevent duplicate diagnostic studies.
- Use information and communication technology (ICT) to facilitate the monitoring and management of older peoples’ disease.
- Move toward patient-centered care.

## 8. Conclusions

A panel of Spanish specialists with vast clinical experience in the management of PCa participated in the ENFOCA2 project, promoted by the Spanish Oncology Genitourinary Group (SOGUG), aiming to discuss and develop updated recommendations for optimizing the care of PCa patients. Improvement of adherence to active surveillance in low-risk groups and evaluation of genetic testing for a better indication of focal therapy are important considerations in localized disease. In locally advanced PCa with PSMA PET/CT-positive lesions in M0 staging on conventional imaging techniques, there is no gold standard regarding therapeutic decisions. In advanced PCa, it is essential to select the most effective first-line treatment because subsequent strategies depend on this choice. Combinations of ARSi and PARPi are emerging alternatives in advanced PCa. In patients older than 70 years, treatment should be individualized after physical and functional geriatric assessment. A multidisciplinary team approach is essential in making decisions and providing personalized care for PCa patients at every stage of the disease. The concept of a comprehensive multidisciplinary approach together with inter-center and/or inter-specialty therapeutic alliances should be implemented in the routine care of patients with PCa.

## Figures and Tables

**Table 1:** Radical and palliative approach in locally advanced disease.

Recommendations	Strength Rating
Radical prostatectomy	
– Offer to patients with cN0 disease as part of multimodal therapy	Weak
Extended pelvic lymph node dissection (ePLND)	
– Perform an ePLND	Strong
Radiotherapy	
– Offer patients with cN0 disease intensity-modulated radiation therapy (IMRT)/volumetric-modulated arc therapy (VMAT) plus image-guide radiation therapy (IGRT), in combination with long-term androgen deprivation therapy (ADT)	Strong
– Offer patients with cN0 disease and good urinary function IMRT/VMAT plus IGRT with brachytherapy boost (either high-dose or low-dose rate), in combination with long-term ADT	Weak
– Offer long-term ADT for at least 2 years	Strong
– Offer IMRT/VMAT plus IGRT to the prostate in combination with long-term ADT and 2 years of abiraterone to cN0M0 patients with ≥2 high-risk factors (cT3-4, Gleason ≥ 8, or PSA ≥ 40 ng/mL)	Strong
– Offer IMRT/VMAT plus IGRT to the prostate plus pelvis in combination with long-term ADT and 2 years of abiraterone to cN1M0 patients	Strong
Therapeutic options outside surgery or radiotherapy – Do not offer whole-gland treatment or focal therapy	Strong

Strong recommendations typically indicate a high degree of evidence quality and/or a favorable balance of benefit to harm and patient preference. Weak recommendations typically indicate availability of lower-quality evidence, and/or equivocal balance between benefit and harm, and uncertainty or variability of patient preference [33].

**Table 2:** Overall survival of different treatment lines in mCRPC patients included in randomized clinical trials.

Treatment [Reference]	Patients	Overall Survival, Months			
		Experimental Arm	Control Arm	Hazard Ratio (95% CI)	*p*-Value
First line					
Docetaxel vs. mitoxantrone [54]	1006	19.2	13.6	0.79 (0.67–0.93)	0.004
Abiraterone vs. placebo [55]	1088	34.7	30.3	0.81 (0.70–0.93)	0.003
Enzalutamide vs. placebo [56]	1717	35.3	31.3	0.77 (0.67–0.88)	<0.001
Second line and successive					
Cabazitaxel vs. mitoxantrone [57]	755	15.1	12.7	0.70 (0.59–0.83)	<0.001
Abiraterone vs. placebo [58]	1195	15.8	11.2	0.74 (0.64–0.86)	<0.001
Enzalutamide vs. placebo [59]	1199	18.4	13.6	0.63 (0.53–0.75)	<0.001
Radium-223 vs. placebo [60]	921	14.9	11.3	0.70 (0.58–0.83)	<0.001

**Table 3:** Criteria for the definition of European Prostate Cancer Centers of Excellence (EPCCE).

Fields	Characteristics/Requirements
1. Clinical step	– Core team
	– Associated services
	– Multidisciplinary approach
	– Diagnostic pathway
	– Treatment pathway
2. Research step	– Internal monitoring of outcomes
	– Clinical data collected through a prespecified database
	– Clinical outcomes should be periodically assessed
	– Prospective trials had to be conducted
3. Educational step	– Structured fellowship programs of 1 year (6 months of research, 6 months of clinics)
4. Quality assurance	– Quality control procedures related to quality assessment of the previous three steps

**Table 4:** Management of older patients with PCa based on recommendations of the International Society of Geriatric Oncology (SIOG).

Elderly patients should be managed according to their individual health status and not according to age
2. Fit elderly patients should receive the same treatment as younger patients on the basis of international recommendations
3. At the initial evaluation, screening for cognitive impairment is mandatory to establish patient competence in making decisions
4. Initial evaluation of health status should use the validated G8 screening tool
5. Abnormal scores on the G8 should lead to a simplified geriatric assessment that evaluates: – Comorbid conditions (Cumulative Illness Score Rating–Geriatrics scale) – Dependence (activities of daily living) – Nutritional status (weight loss)
6. When patients are frail or disabled or have severe comorbidities, a comprehensive geriatric assessment is needed

## Data Availability

Data sharing is not applicable. No new data were created or analyzed in this study.
